# Increased malaria parasitaemia among adults living with HIV who have discontinued cotrimoxazole prophylaxis in Kitgum district, Uganda

**DOI:** 10.1371/journal.pone.0240838

**Published:** 2020-11-11

**Authors:** Philip Orishaba, Joan N. Kalyango, Pauline Byakika-Kibwika, Emmanuel Arinaitwe, Bonnie Wandera, Thomas Katairo, Wani Muzeyi, Hildah Tendo Nansikombi, Alice Nakato, Tobius Mutabazi, Moses R. Kamya, Grant Dorsey, Joaniter I. Nankabirwa

**Affiliations:** 1 Clinical Epidemiology Unit, College of Health Sciences, Makerere University, Kampala, Uganda; 2 Department of Pharmacy, College of Health Sciences, Makerere University, Kampala, Uganda; 3 Department of Internal Medicine, College of Health Sciences, Makerere University, Kampala, Uganda; 4 Infectious Diseases Research Collaboration, Kampala, Uganda; 5 Division of Infectious Diseases, Department of Medicine, University of California San Francisco, San Francisco, California, United States of America; Universidade Nova de Lisboa Instituto de Higiene e Medicina Tropical, PORTUGAL

## Abstract

**Background:**

Although WHO recommends cotrimoxazole (CTX) discontinuation among HIV patients who have undergone immune recovery and are living in areas of low prevalence of malaria, some countries including Uganda recommend CTX discontinuation despite having a high malaria burden. We estimated the prevalence and factors associated with malaria parasitaemia among adults living with HIV attending hospital outpatient clinic before and after discontinuation of CTX prophylaxis.

**Methods:**

Between March and April 2019, 599 participants aged 18 years and above, and attending Kitgum hospital HIV clinic in Uganda were enrolled in a cross study. A standardized questionnaire was administered and physical examination conducted. A finger-prick blood sample was collected for identification of malaria parasites by microscopy. The prevalence of parasitaemia was estimated and compared among participants on and those who had discontinued CTX prophylaxis, and factors associated with malaria parasitaemia assessed.

**Results:**

Of the enrolled participants, 27 (4.5%) had malaria parasites and 452 (75.5%) had stopped CTX prophylaxis. Prevalence of malaria parasitaemia was significantly higher in participants who had stopped CTX prophylaxis (5.5% versus 1.4% p = 0.03) and increased with increasing duration since the discontinuation of prophylaxis. Compared to participants taking CTX, those who discontinued prophylaxis for 3–5 months and >5 months were more likely to have malaria parasites (adjusted prevalence ratio (aPR) = 1.64, 95% CI 0.37–7.29, p = 0.51, and aPR = 6.06, 95% CI 1.34–27.3, P = 0.02). Low CD4 count (< 250cells/mm^3^) was also associated with increased risk of having parasites (aPR = 4.31, 95% CI 2.13–8.73, p <0.001).

**Conclusion:**

People from malaria endemic settings living with HIV have a higher prevalence of malaria parasitaemia following discontinuation of CTX compared to those still on prophylaxis. The risk increased with increasing duration since discontinuation of the prophylaxis. HIV patients should not discontinue CTX prophylaxis in areas of Uganda where the burden of malaria remains high. Other proven malaria control interventions may also be encouraged in HIV patients following discontinuation of CTX prophylaxis.

## Introduction

Malaria and HIV are among the major health problems affecting developing countries [1, 2]. Together, they account for more than 2 million deaths a year globally [3]. The overlap of HIV and malaria in the sub Saharan region, including Uganda, means that co-infection with both diseases is common [4]. The impact of the malaria-HIV coinfection is most apparent in areas with HIV epidemics and stable malaria transmission [3]. HIV infection can increase the risk and severity of malaria infection, and may facilitate higher rates of malaria transmission by increasing parasite burden [5, 6]. On the other hand, malaria infections in HIV patients induce a non-specific lymphopenia and a decrease of CD4 cell count which is associated with increasing levels of HIV RNA replication [7, 8].

In 2001, the WHO recommended cotrimoxazole (CTX) as part of the minimum package of care for PLHIV in Africa [9]. This recommendation followed findings that demonstrated that CTX prophylaxis significantly reduces HIV related mortality, bacterial infections, malaria and related hospital admissions [10, 11]. Uganda adopted this recommendation in 2005 as part of their basic HIV preventive care package [12]. Although CTX prophylaxis has been associated with significant benefits, its use has been associated with increases in cost of care, risk of haematological toxicity, hypersensitivity skin reactions and pill burden [13]. Following the wide spread use of Anti-retroviral Therapy (ART), studies have shown a reduction in the risk of opportunistic infections in patients who have improved immune function [14]. Based on these findings, and in order to reduce the risks of adverse events, the WHO recently revised its guidelines and currently recommends that CTX prophylaxis may be discontinued for adults with HIV infection who are clinically stable on ART, with evidence of immune recovery and viral suppression [15]. The new guidelines also recommend that CTX prophylaxis should be continued regardless of CD4 cell count or WHO clinical stage in settings where malaria and/or severe bacterial infections are prevalent [15]. Unfortunately, due to the high costs of CTX, some malaria endemic countries including Uganda have recommended the discontinuation of CTX in clinically stable patients on ART [16].

One trial in Uganda assessed the safety of discontinuing CTX in stable patients on ART and found that patients were at increased risk of malaria [17], however, data on the effect of the CTX prophylaxis discontinuation on malaria burden in routine clinical practice is still limited. We determined the prevalence and assessed the factors associated with malaria parasitaemia among adults living with HIV attending the Kitgum hospital HIV clinic six months following the roll out of the policy on CTX discontinuation in Uganda.

## Materials and methods

### Study design and setting

A cross-sectional study was conducted between March and April 2019. The study was carried out at the HIV outpatient clinic of Kitgum Hospital, the largest public health facility in Kitgum district that offers health care services free of charge to its clients with support from the government of Uganda. The HIV clinic at the hospital receives patients from across the Acholi sub-region (including the districts of Kitgum, Abim, Agago, Kabong, Kotido, Lamwo, Nwoya, Pader) and some from South Sudan. During the course of the study, the HIV clinic was open four days a week (Monday to Thursday) from 8am to 5pm, close to 50 patients were seen per clinic day, and had about 3,000 people living with HIV registered.

According to the 2018/2019 Uganda malaria indicator survey, the prevalence of microscopic parasitaemia in children under five years in Kitgum district is estimated at 12% [18, 19], with two peak transmission seasons following the rainy seasons of May and August [20]. Malaria control interventions in the district include; Indoor residual spraying (IRS); this was initiated in 2009 using alpha-cypermethrin. To date, there have been 7 rounds of IRS in the district with the last round done in 2017 using Actellic. Use of long-lasting insecticide treated bed nets; the district has had 2 rounds of free bed-net distribution campaigns in 2013/14 and 2017 [21]. Other interventions include: treatment of cases with artemisinin combined therapy and intermittent preventive treatment with sulfadoxine and pyrimethamine during pregnancy.

### Study participants and data collection

We consecutively enrolled 599 people living with HIV who attended the Kitgum hospital HIV clinic. Participants were included in the study if they: 1) visited the clinic during the study period, 2) were aged 18 years and above, 3) were either on or off CTX for at least 3 months, 4) provided written informed consent. Participants were excluded if they declined to have a finger prick blood sample to be collected. A pre-tested questionnaire was administered by study staff to all enrolled participants including data on participant socio-demographics (such as age, sex, education level, marital status, employment status, number of members in the house hold, religion among others) and clinical characteristics (CD4 count, viral load status, time on ART and duration since CTX withdrawal). Data on clinical characteristics was abstracted from the patient’s files at the time of the interview. The questionnaire was pretested among the first 30 participants of the study. The results of the participants among whom the pre-test was done were not included in the analysis. A finger prick blood smear was collected onto a frosted slide which was labelled with a participant’s identification number. All smears were air dried, and stored in slide boxes, and transferred to the molecular research laboratory of the Infectious Diseases Research Collaboration for staining and reading.

### Laboratory evaluations

Thick blood smears were stained with 2% Giemsa for 30 minutes and evaluated for the presence malaria parasites. Parasite densities were calculated by counting the number of malaria parasites per 200 leukocytes (or per 500, if the count was less than 10 parasites per 200 leukocytes), assuming a leukocyte count of 8,000/μl. A thick blood smear was considered negative if examination of 100 high power fields revealed no malaria parasites. For quality control, all slides were read by a second microscopist and a third microscopist settled any discrepant readings [22].

### Sample size

The study was designed to test the hypothesis that participants who had discontinued CTX prophylaxis for at least three months would have a higher prevalence of malaria parasitaemia than those on prophylaxis. Using the sample size of two proportions and setting the prevalence of parasitaemia in the CTX prophylaxis group at 4% and that in the group that had discontinued prophylaxis at 13.2% with the estimated proportions of participants being 53% and 47% on CTX and off CTX respectively [23]. We needed a total of 511 participants to test the hypothesis at 95% confidence level and power of 80%.

### Statistical analysis

All data were collected using standardized questionnaires and double-entered using Microsoft Access (Microsoft Corporation, Redmond, Washington, USA). Analyses were performed using Stata, version 14 (Stata Corporation, College Station, Texas, USA). Descriptive characteristics included proportions for categorical variables, and mean (SD) or median (IQR) values for continuous variables. The prevalence of malaria parasitaemia was defined as the number of individuals with a positive malaria smear divided by the total number of participants tested, and was stratified by whether they were on prophylaxis, had discontinued prophylaxis between 3–5 months or had discontinued prophylaxis for more than 5 months. The prevalence of parasitaemia among those on CTX prophylaxis was compared to the prevalence among those who had discontinued CTX prophylaxis using Pearson’s chi-square test. To test for difference in parasite density between groups (CD4 count, viral load), Wilcoxon rank-sum (Mann-Whitney) test was used. Univariable and multivariate associations between malaria infection and potential associated factors were assessed using robust (modified) Poisson regression. Measures of association were reported as prevalence ratios with their 95% confidence intervals (CI). Given the relatively large sample size and in order to eliminate all potential confounders, a saturated model was used in the multivariate analysis and p-value of 0.05 was considered significant. Sensitivity analysis comparing the model that only included significant outcomes to the saturated model showed no difference in the estimates generated.

### Ethical approval

Ethical approval was obtained from the Makerere University School of Medicine Research and Ethics Committee (#REC REF 2019–061). Administrative permission to conduct the study was obtained the Kitgum hospital board and written informed consent was obtained from all the research participants.

## Results

### Characteristics of the study population

**Fig 1:**
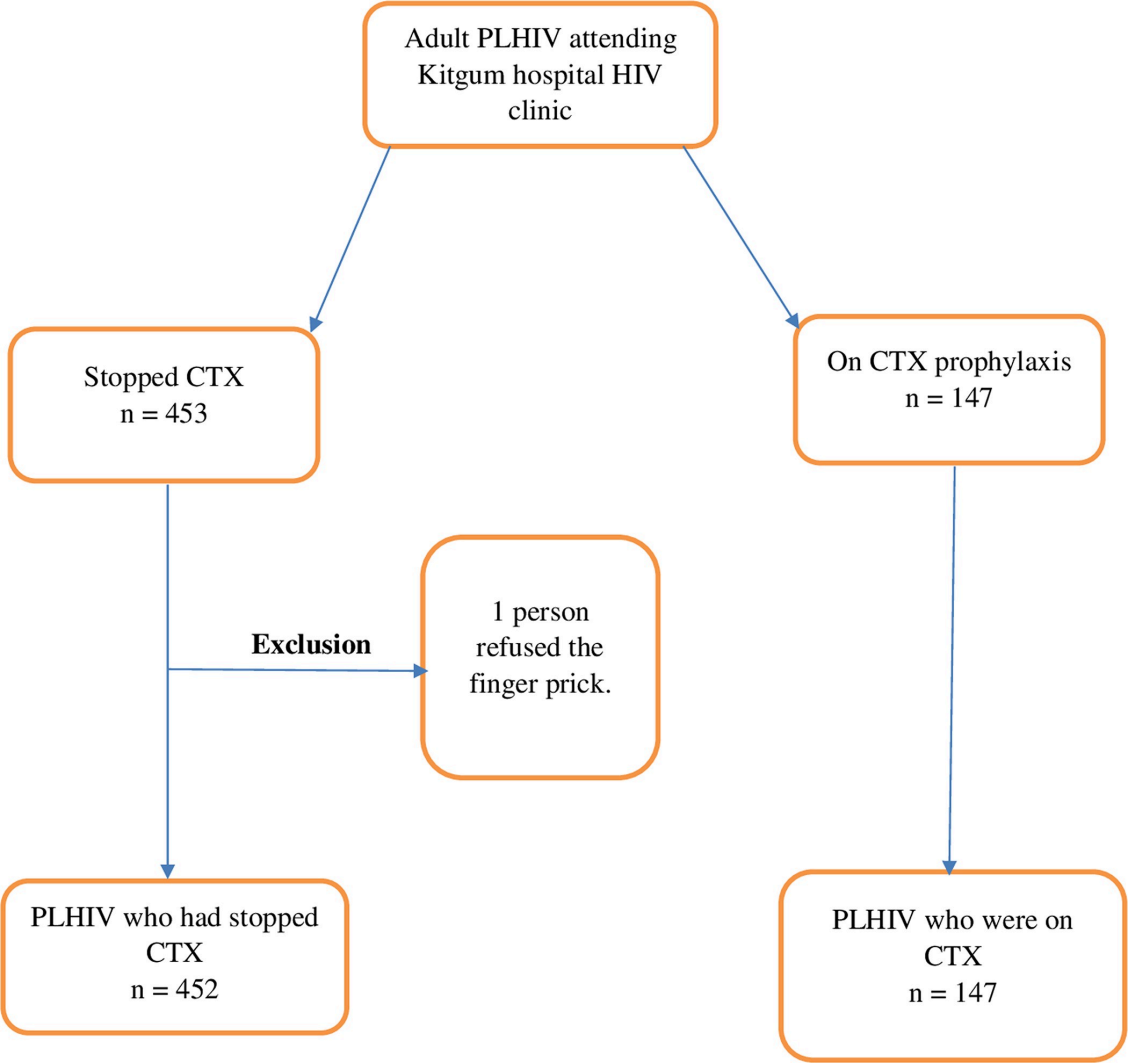
Participant recruitment to the study.

Out of 600 people living with HIV screened, 599 (99.8%) were enrolled in the study. Only one patient was screened but not enrolled because he declined to provide consent as he did not want to have a finger-pick blood sample collected (Fig 1). All the participants of this study were asymptomatic. The majority of participants were female (67.5%) and the median age of participants was 39 years (interquartile range 33–47) (Table 1).

**Table 1:** Characteristics of 599 people enrolled in the study.

	No CTX	CTX	P value
	n (%)	n (%)	
**Number of participants**	452	147	
**Age** in years, median (IQR)	40 (33, 48)	35 (29, 43)	<0.001
**Sex**			
Female	315 (69.7)	88 (59.9)	0.028
**Education**			
None	95 (21.0)	24 (16.3)	0.316
Primary	239 (52.9)	72 (49.0)	0.312
Secondary	95 (26.1)	40 (34.7)	0.118
Tertiary	23 (5.1)	11 (7.5)	0.862
**Marital status**			
Married	263 (75.8)	84 (24.2)	<0.001
**Employment**			
Unemployed	323 (71.5)	100 (68.0)	0.418
**Number of members in Household**			
≤ 5	232 (51.3)	83 (56.5)	0.273
**Time off cotrimoxazole**			
Off CTX 3–5 months	347 (76.8)		
Off CTX for more than 5 months	105 (23.2)		
**IRS (in the last one year)**			
Yes	409 (90.5)	138 (93.9)	0.203
**Bed-net use (night before survey)**			
Yes	363 (80.3)	128 (87.1)	0.062
Baseline CD4 count*			
≥250	354 (86.1)	83 (83.0)	0.356
Viral load status**			
Not detectable	423 (94.6)	58 (55.2)	<0.001
ART regimen***			
1st line	446 (98.9)	139 (94.6)	0.002

* Assessed in 511 participants with baseline CD4 count records

** Assessed in 552 participants with viral load status results

*** Assessed in 598 participants with known ART regimen.

Of the enrolled participants, 491 (82.1%) reported having used an insecticide treated net (ITN) the night before the survey, and 547 (91.3%) reported having had their house sprayed with an insecticide within the last one year. Most participants (97.8%) were still on the first line of ART treatment. Of the 599 participants enrolled, 452 (75.5%) had discontinued CTX prophylaxis. Mean duration since CTX discontinuation was 4.5 months (1.2). Participants who had discontinued CTX prophylaxis were older and were more likely to have undetectable viral load than those on prophylaxis. The overall number of participants that had CD4 results in this study was 85.3% (511/599). Of the 147 participants still taking CTX prophylaxis, 68.0% (100) had CD4 results. Of the 100 participants on CTX prophylaxis and having CD4 results, 83.0% (83/100) had a CD4 count ≥250cells/mm^3^. On the other hand, 411/452 (90.9%) participants who had stopped CTX had CD4 results and of those, 86.1% (354/411) had a CD4 count <250cells/mm^3^. In addition, 92.2% (552/599) of the participants in this study had viral load results. Of the 105 participants on prophylaxis and with viral load test results, 55.2% (58/105) had a non-detectable viral load result. Similarly, 5.4% (24/447) of the participants who had a detectable viral load had stopped CTX prophylaxis.

### Prevalence of malaria parasitaemia by CTX prophylaxis groups

**Fig 2:**
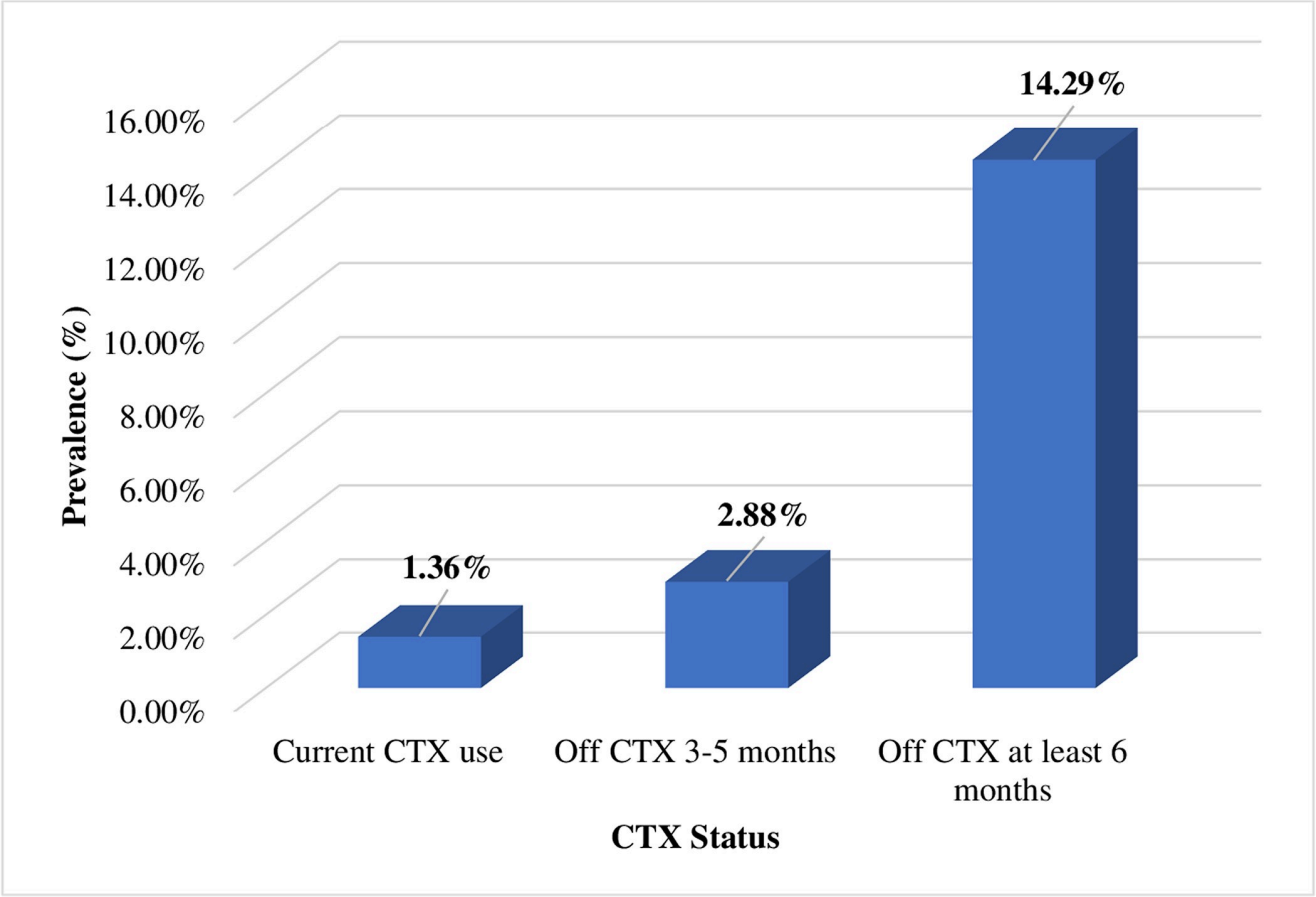
Prevalence of malaria parasitaemia stratified by cotrimoxazole status.

The overall prevalence of malaria parasitaemia was 4.5% (27/599). On stratifying by CTX prophylaxis, the prevalence of parasitaemia increased with increasing duration since discontinuation of prophylaxis. Participants who were still on CTX prophylaxis had the lowest prevalence of parasitaemia (1.4%), followed by those who had stopped for 3–5 months (2.9%). Participants who had discontinued prophylaxis for more than 5 months had the highest prevalence of parasitaemia of 14.3% (Fig 2). The lowest recorded parasite density among the participants of this study was 400 parasites/*μ*L of blood while the highest was 8,500 parasites /*μ*L of blood. The mean parasite density was 1,790 parasites /*μ*L of blood. The median (IQR) parasite density was 1,200 (1,000–1,800) parasites /*μ*L.

### Factors associated with malaria parasitaemia in the study population

Factors that were significantly associated with malaria parasitaemia among the study participants included status of CTX prophylaxis and CD4 count (Table 2). The risk of having malaria parasites was associated with increased duration since CTX discontinuation. Compared to those on CTX prophylaxis, the prevalence of parasitaemia was higher in participants that had been off CTX prophylaxis for 3–5 months but this did not reach statistical significance (adjusted risk ratio [aPR] = 1.64, 95% confidence interval [CI] 0.37–7.29, p = 0.51), however, those off prophylaxis for at least 6 months had a much higher prevalence of parasitaemia (aPR = 6.06 95% CI 1.34–27.3, p = 0.02). In addition, participants with CD4 count less than 250 cells/mm^3^ were more likely to have parasites than participants with CD4 count of 250 cells/mm^3^ or higher (aPR = 4.31 95% CI 2.13–8.73, p<0.001).

**Table 2:** Factors associated with malaria parasitaemia among participants in whom cotrimoxazole prophylaxis was discontinued.

Variable	Categories	Percentage Parasitaemia (%)	Unadjusted analysis		Adjusted analysis	
			PR (95% CI)	P value	PR (95% CI)	P value
CTX status	On for at least 3 months	2/147 (1.4%)	reference group		reference group	
	Off for 3–5 months	10/347 (2.9%)	2.12 (0.47–9.56)	0.33	1.64 (0.37–7.29)	0.51
	Off for more than 5 months	15/105 (14.3%)	10.5 (2.45–45.0)	0.002	6.06 (1.34–27.3)	0.02
IRS use in the previous 1 year	Yes	21/547 (3.8%)	reference group		reference group	
	No	6/52 (11.5%)	3.01 (1.27–7.12)	0.01	1.35 (0.46–3.93)	0.59
Bed-net use the previous night	Yes	16/491 (3.3%)	reference group		reference group	
	No	11/108 (10.2%)	3.13 (1.49–6.55)	0.003	2.18 (0.70–6.75)	0.18
CD4 count *	≥ 250	14/437 (3.2%)	reference group		reference group	
	<250	11/74 (14.9%)	4.64 (2.19–9.83)	<0.001	4.31 (2.13–8.73)	<0.001
Age (per 1-year increase)		N/A	1.01 (0.98–1.03)	0.71	0.99 (0.96–1.03)	0.66
Gender	Male	8/196 (4.1%)	reference group		reference group	
	Female	19/403 (4.7%)	1.16 (0.51–2.59)	0.73	0.92 (0.32–2.66)	0.88
Education	Beyond primary	5/169 (3.0%)	reference group		reference group	
	Primary	15/311 (4.8%)	1.63 (0.60–4.41)	0.34	1.42 (0.50–4.01)	0.51
	None	7/119 (5.9%)	1.99 (0.65–6.12)	0.23	1.43 (0.32–6.47)	0.64
Marital status	Single	11/252 (4.4%)	reference group		reference group	
	Married	16/347 (4.6%)	1.06 (0.50–2.24)	0.89	1.07 (0.45–2.54)	0.88
Number of household members	≤ 5	13/315 (4.1%)	reference group		reference group	
	> 5	14/284 (4.9%)	1.19 (0.57–2.50)	0.64	1.01 (0.47–2.19)	0.97

In the unadjusted analyses, no IRS use in the previous 1 year (PR = 3.01, 95%CI 1.27–7.12, p = 0.01) and not using an ITN the previous night (PR = 3.13, 95%CI 1.49–6.55, p = 0.003) were associated with an increased risk of parasitaemia, but these associations were not statistically significant in the adjusted analysis.

**Fig 3:**
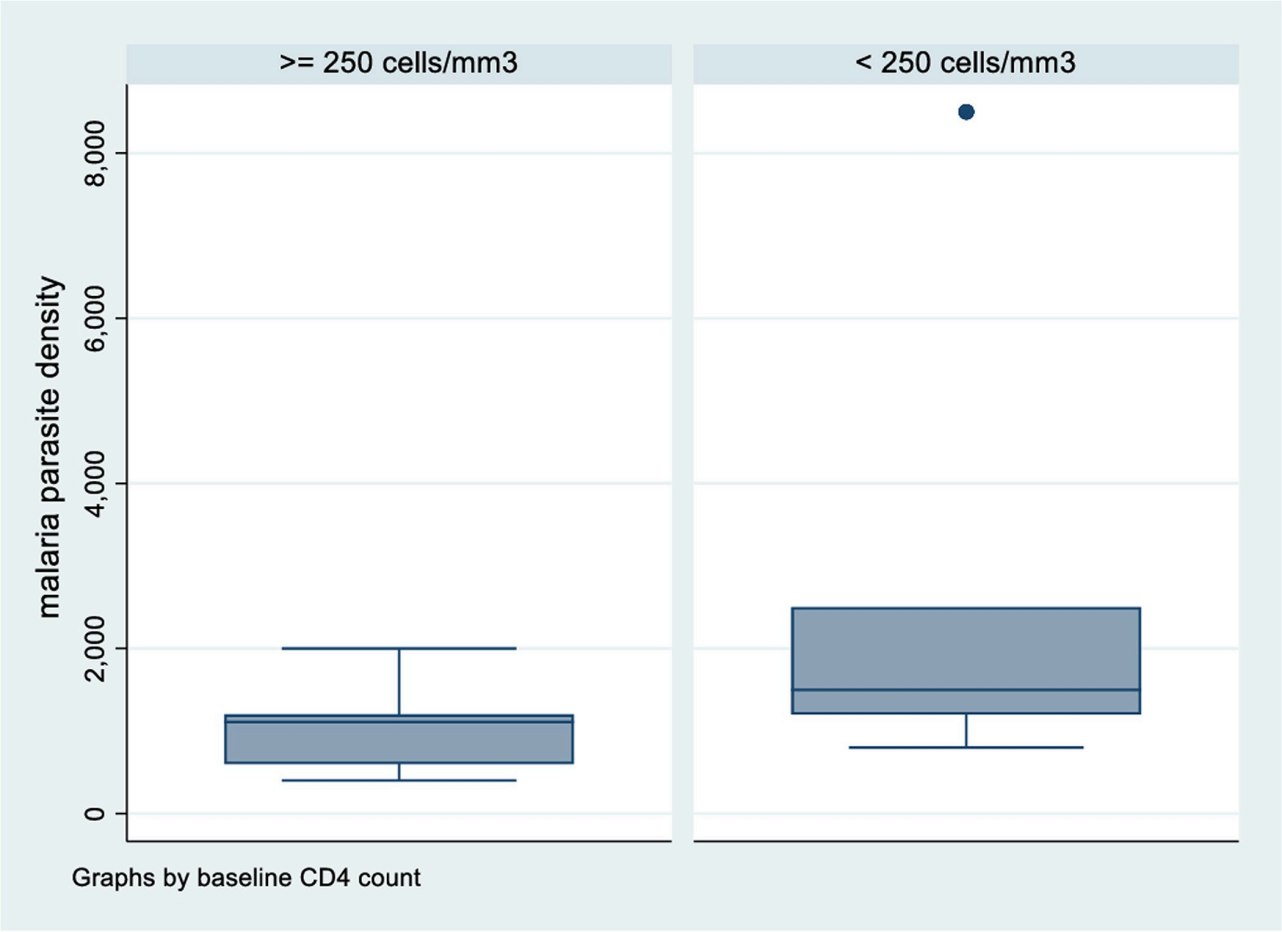
Malaria parasite density variation with CD4 cell count.

**Fig 4:**
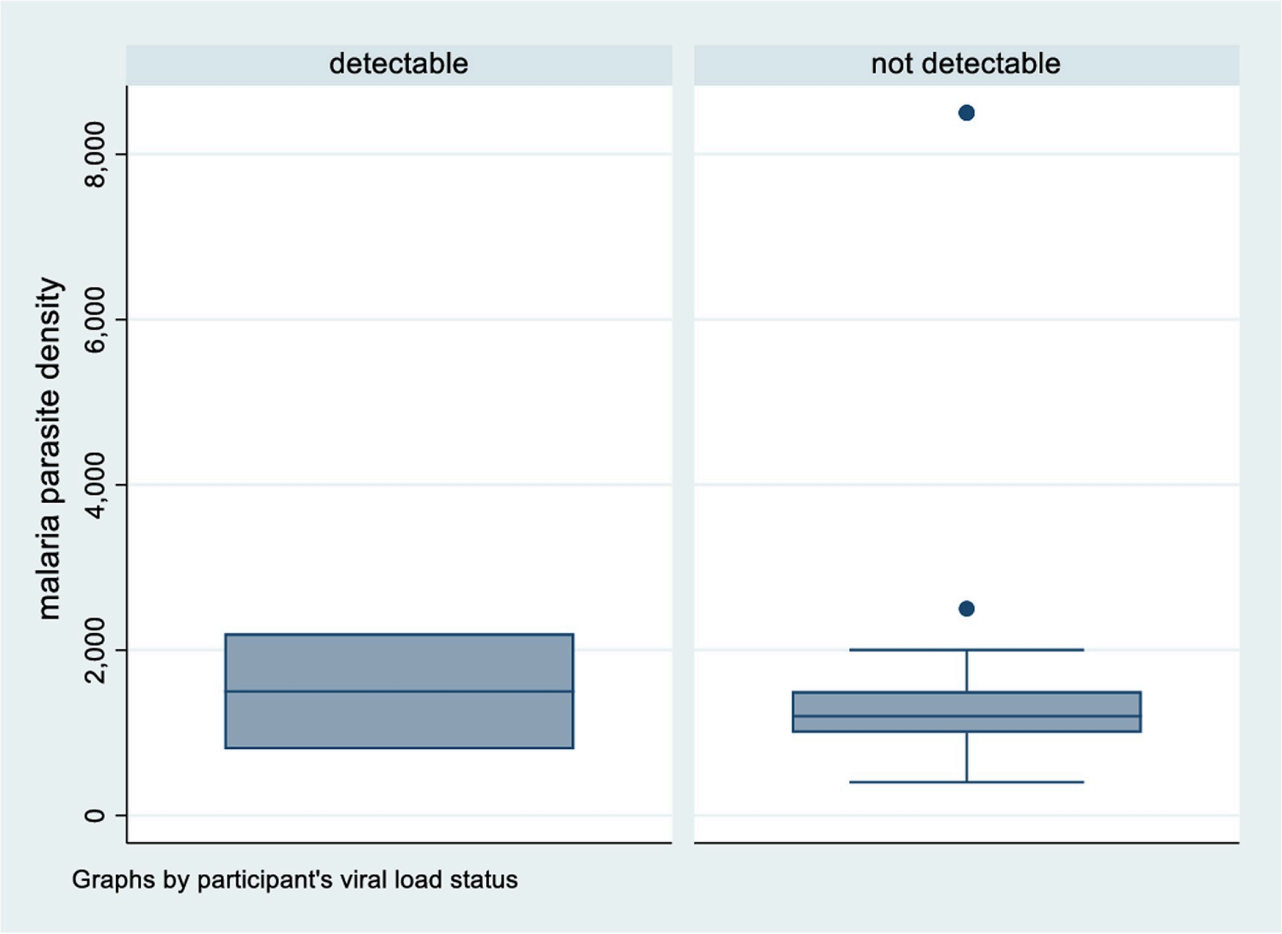
Malaria parasite density variation with viral load test result.

The participating individuals’ CD4 counts were also analysed against parasite density. Findings from the analysis suggest that participants with low CD4 counts (<250 cells/mm^3^) had higher parasite densities [(median (IQR) = 1,500 (1,200–2,500)] when compared to participants with CD4 counts of 250 cells/mm^3^ [(median (IQR) = 1,110 (600–1,200)], p value = 0.012 (Fig 3). There was no significant difference in parasite densities between participants who had a detectable viral load [(median (IQR) = 1,500 (800–2,200)] in comparison to participants with a non-detectable viral load [(median (IQR) = 1,200 (1,000–1,500)], p value = 0.913 (Fig 4). The results also suggest that malaria parasite densities increased with increase in the number of months off CTX prophylaxis but the association was not statistically significant (p value = 0.389).

## Discussion

This study explored the prevalence and factors associated with malaria parasitaemia among people living with HIV and attending Kitgum Hospital HIV clinic six months following the recent policy recommendation to discontinue CTX prophylaxis in Uganda in September 2018 [16]. Findings from this study show that the prevalence of malaria parasitaemia was relatively high in the population and increased with increasing duration since discontinuation of CTX prophylaxis. In addition, low CD4 count (less than 250 cells/mm^3^) was associated with increased risk of having parasites in the study population.

Although the malaria prevalence estimated from this study population is comparatively lower than the 12% prevalence in the district [24] and previously estimated prevalences in the HIV populations in Uganda [25–27] and elsewhere in the world in Cameroon and South West Nigeria respectively [28, 29], we observed a significant increase in the risk of infection with the increasing duration since CTX discontinuation. Participants that had discontinued CTX prophylaxis for six or more months were apparently more likely to have parasites compared to those still on prophylaxis. The observed increase in parasite prevalence in the groups that had discontinued CTX prophylaxis is likely explained by the loss of the protective effect that CTX offers against malaria infections when the prophylaxis is discontinued.

CTX, although grouped under antibiotics has been shown to be effective in the treatment and prevention of malaria infections irrespective of whether they have HIV or not with evidence from studies done in Côte d'Ivoire, Uganda and Nigeria [10, 11, 30]. Malaria parasitaemia in HIV infection is worrisome given that the patients are already immune compromised, and HIV and *Plasmodium* interrelate with the host's immune system, which may result in HIV replication and thus increased morbidity and mortality [31]. To maintain the benefits of CTX, in 2014 when the recommendations on CTX prophylaxis were revised, the WHO specified that CTX may be discontinued in adults on ART following HIV virologic suppression but this should be in settings where malaria and/or severe bacterial infections are not prevalent [15]. Unfortunately, some low-income countries including Uganda have adopted the new recommendations despite having a high malaria burden [16]. The findings from our study highlight the importance of maintaining CTX prophylaxis in adult patients living with HIV in high malaria endemic countries even when they have achieved viral suppression.

We also note that a low CD4 count (<250 cell/mm^3^) was associated with a fourfold increase in risk of having malaria parasites independent of whether CTX was discontinued. Similarly, participants with low CD4 counts had significantly increasing parasite densities in comparison to participants with higher CD4 counts. From this study however, it is unclear whether the low CD4 is the risk factor or the effect of having the malaria parasites. Both HIV and malaria destroy important cells required for proper immunological functioning of the body [32] and interrelate with the host's immune system, yielding complex activation of immune cells which leads to dysregulated levels of antibody and cytokine generation [33–35]. This means that malaria/HIV co-infections could worsen the immune response to both diseases due to enhanced T-cell activation and may explain the low CD4 count in patients coinfected with both diseases. However, some studies in malaria endemic regions have reported the risk of malaria to be higher in patients with low CD4 cell counts [36] which is the most probable explanation of what is observed in this study. Indeed, a high CD4 is one of the recognized factors highlighted when considering the discontinuation of CTX prophylaxis in an HIV patient, with a recommendation to continue CTX prophylaxis in HIV patients with a CD4 count of ≤350 cells/mm^3^ among other considerations [15]. Immune deficiency syndrome due to HIV depletion of CD4 T-cells may also explain the high parasite density among this population. HIV/AIDS not only induces depletion of CD4 T-cells but also reduces CD8 T-cells leading to downmodulation, reduction in T-cell subpopulation and defective cell mediated immunity against any microbial infection [37, 38].

Uganda has rolled out several effective malaria control interventions including IRS, use of ITNs and malaria case management with an ACT. Our study findings show that participants who reported using a bed net the night before the survey and those who had IRS within the last one year were more likely to be protected from malaria infections than those not using protective measures, although the findings were not significant at multivariate analysis. Bed nets protect against malaria by providing a barrier against mosquito bites and when treated with insecticides kill the mosquitos on contact. When effectively and consistently used, bed-nets have been shown to reduce the risk of malaria infection in the populations living in malaria endemic countries [39, 40]. Previous literature particularly in Uganda showed that although many people possess bed-nets, majority do not consistently use them [41]. On the other hand, IRS involves the application of residual insecticides on the vector resting places, and where applied has been associated with reduction in the mosquito densities, malaria incidence, parasite prevalence, birth outcomes and malaria transmission [42, 43]. Although not routinely practiced, our results suggest that provision of bed-nets and promoting their use as well as using IRS in HIV patients reduce the risk of malaria in this population, and may become strong control tools in HIV patients who are discontinuing CTX prophylaxis.

Our study was not without limitations. The period for data collection was limited to three months and at one HIV clinic. Given the short duration of the study, we did not capture how the estimates change in the different malaria seasons and with changing patient health seeking behaviours. We however believe that even when the strength of association may differ, the conclusion would have been the same had the study period been extended. Furthermore, this study was designed in a cross-sectional fashion hence making it impossible to establish a causal relationship because information about the exposure and the outcome was collected at the same point in time. In addition, the study used microscopy for the detection of the parasites and may have missed sub microscopic infection that would have been picked up by the more sensitive molecular methods [44]. Despite the low sensitivity of microscopy, we were able to come to biologically plausible conclusions and are confident that a more sensitive test would come to stronger conclusions of our study findings.

In conclusion, our study findings indicate that people with HIV and living in malaria endemic settings who discontinue CTX prophylaxis have a higher risk of having malaria parasitaemia than those on prophylaxis, and the risk increases with increasing duration since discontinuation of the treatment. We recommend that HIV infected patients living in areas of high malaria burden should not stop CTX prophylaxis even following immune recovery. There is also need to provide and encourage the use of other proven malaria control interventions in HIV patients when CTX prophylaxis is discontinued in order to reduce their risk of morbidity and mortality due to malaria.
